# Cutaneous nociceptors lack sensitisation, but reveal μ-opioid receptor-mediated reduction in excitability to mechanical stimulation in neuropathy

**DOI:** 10.1186/1744-8069-8-81

**Published:** 2012-11-02

**Authors:** Yvonne Schmidt, Dominika Labuz, Paul A Heppenstall, Halina Machelska

**Affiliations:** 1 Klinik für Anästhesiologie und operative Intensivmedizin, Freie Universität Berlin, Charité-Universitätsmedizin Berlin, Campus Benjamin Franklin, Berlin, D-12203, Germany; 2 Current address: EMBL Monterotondo, Adriano Buzzati-Traverso Campus, Monterotondo, 00016, Italy

**Keywords:** Neuropathic pain, Nociceptors, Mechanical sensitivity, Opioids

## Abstract

**Background:**

Peripheral nerve injuries often trigger a hypersensitivity to tactile stimulation. Behavioural studies demonstrated efficient and side effect-free analgesia mediated by opioid receptors on peripheral sensory neurons. However, mechanistic approaches addressing such opioid properties in painful neuropathies are lacking. Here we investigated whether opioids can directly inhibit primary afferent neuron transmission of mechanical stimuli in neuropathy. We analysed the mechanical thresholds, the firing rates and response latencies of sensory fibres to mechanical stimulation of their cutaneous receptive fields.

**Results:**

Two weeks following a chronic constriction injury of the saphenous nerve, mice developed a profound mechanical hypersensitivity in the paw innervated by the damaged nerve. Using an *in vitro* skin-nerve preparation we found no changes in the mechanical thresholds and latencies of sensory fibres from injured nerves. The firing rates to mechanical stimulation were unchanged or reduced following injury. Importantly, μ-opioid receptor agonist [D-Ala^2^,*N*-Me-Phe^4^,Gly^5^]-ol-enkephalin (DAMGO) significantly elevated the mechanical thresholds of nociceptive Aδ and C fibres. Furthermore, DAMGO substantially diminished the mechanically evoked discharges of C nociceptors in injured nerves. These effects were blocked by DAMGO washout and pre-treatment with the selective μ-opioid receptor antagonist Cys^2^-Tyr^3^-Orn^5^-Pen^7^-amide. DAMGO did not alter the responses of sensory fibres in uninjured nerves.

**Conclusions:**

Our findings suggest that behaviourally manifested neuropathy-induced mechanosensitivity does not require a sensitised state of cutaneous nociceptors in damaged nerves. Yet, nerve injury renders nociceptors sensitive to opioids. Prevention of action potential generation or propagation in nociceptors might represent a cellular mechanism underlying peripheral opioid-mediated alleviation of mechanical hypersensitivity in neuropathy.

## Background

Mechanical hypersensitivity is a common consequence of peripheral nerve damage (e.g. compression, stretch or amputation). It includes dynamic (pain in response to light stroking) and static (pain in response to pressure) subtypes, both found in patients suffering from neuropathic pain [1,2]. Similarly, behavioural mechanosensitivity is often reported in animal models of peripheral neuropathic pain [3]. Elucidating the underlying mechanisms requires examination of the relationships between enhanced pain intensity to mechanical stimuli and the function of primary afferent neurons.

Sensitisation to heat manifested by increased discharges and lowered thresholds in high threshold primary afferent neurons (nociceptors) was frequently found under inflammatory and neuropathic conditions [4-7]. However, there are controversial data on mechanical sensitisation. Following inflammation, nociceptors revealed increased firing or lowered mechanical thresholds in some [8-10], but not in other reports [4,5,11-13]. Notably, only few studies examined mechanical sensitivity of cutaneous nociceptors following nerve trauma, reporting higher discharges and unaltered, decreased or increased thresholds [6,7,14,15].

Opioids are undoubtedly the most efficacious analgesics for severe postoperative and cancer pain. However, their satisfactory control of neuropathic pain is limited by distressing side effects. These include nausea, dysphoria, physical dependence and addiction mediated by opioids via μ-, δ- or κ-opioid receptors in the central nervous system (CNS) [16]. Interestingly, opioids can decrease pain devoid of CNS adverse effects through activation of opioid receptors on primary afferent neurons in somatic inflammatory pain [17]. Moreover, behavioural studies revealed that immune cell-derived and exogenously applied opioids acting at their peripheral receptors alleviate mechanical hypersensitivity in animal models of neuropathic pain [18-27]. Also, a clinical pilot trial reported attenuation of neuropathic pain after peripherally applied morphine in patients [28]. Despite substantial behavioural evidence on peripheral opioid-mediated reduction of mechanical hypersensitivity following nerve damage, the underlying neuronal mechanisms are unknown yet.

Our goal was to elucidate whether activation of peripheral μ-opioid receptors can directly reduce neuropathy-induced mechanical excitability of primary afferent neurons. In a mouse model of neuropathic pain, a chronic constriction injury (CCI) of the saphenous nerve, we examined the fibres’ thresholds, firing rates and response latencies to mechanical stimulation of their cutaneous receptive fields.

## Results

### *In vivo* mechanical hypersensitivity following injury of the saphenous nerve

**Figure 1:**
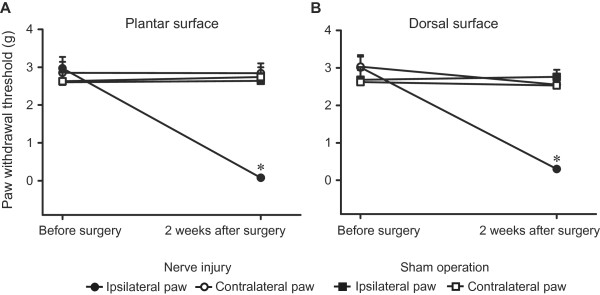
***In vivo *mechanical hypersensitivity following nerve injury.** Measurements were performed with von Frey hairs applied to the plantar (**A**) and dorsal (**B**) surfaces of hind paws, before and 2 weeks after CCI or sham operation on the saphenous nerve. *P < 0.05, compared to thresholds before injury, thresholds of contralateral paws of CCI animals, and of both hind paws of sham-operated animals (2-way RM ANOVA, Bonferroni *t* test). Data are expressed as means ± SEM. N = 6 mice per group.

Two weeks after CCI of the saphenous nerve mice developed a profound mechanical hypersensitivity. This was manifested by significantly lower thresholds of both the plantar and dorsal surface of hind paws innervated by the damaged nerves as compared to the thresholds of contralateral paws, of both hind paws in sham-operated animals, and to the thresholds before injury (*P* < 0.05). There were no significant differences in von Frey thresholds in paws contralateral to the CCI and in hind paws of sham-operated mice (*P* > 0.05; Figure 1). Thus, mechanical hypersensitivity developed in the saphenous nerve territory *in vivo*.

### The impact of nerve injury on peripheral sensory fibre responses to mechanical stimulation

**Figure 2:**
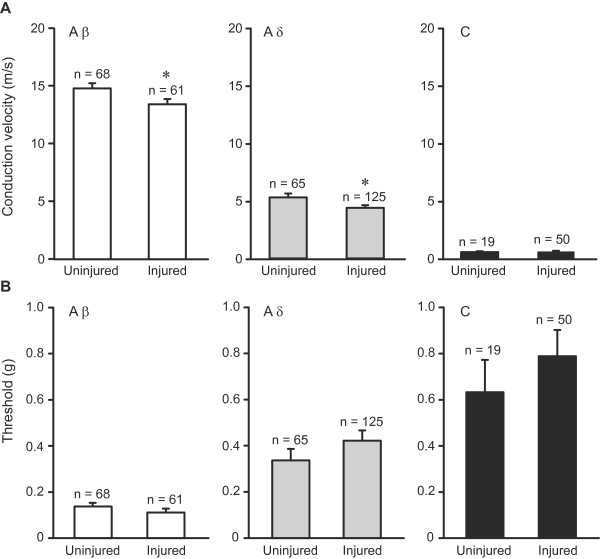
**Conduction velocity and mechanical thresholds of sensory fibres following nerve injury.** (**A**) Conduction velocities of Aβ and Aδ, but not of C fibres, were slightly decreased in injured nerves (*P < 0.05 and *P* > 0.05, respectively; Mann–Whitney test). (**B**) Mechanical von Frey thresholds of sensory fibres were not altered by nerve injury (*P* > 0.05; Mann–Whitney test). In uninjured nerves, the number of fibres from sham-operated and nonoperated nerves is as follows: Aβ fibres (18 and 50), Aδ fibres (12 and 53), and C fibres (5 and 14). All data are expressed as means ± SEM. N, number of fibres.

**Figure 3:**
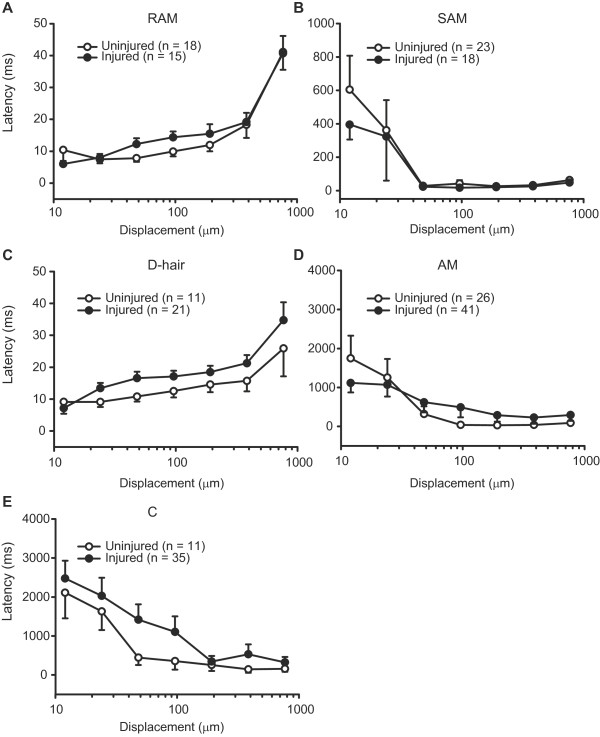
**Mechanical latency of sensory fibres following nerve injury.** Latencies of RAM and SAM (Aβ), D-hair and AM (Aδ), and C fibres to nanomotor stimulation were not significantly altered by nerve injury (*P* > 0.05; 2-way RM ANOVA) (**A-E**). In uninjured nerves, the number of fibres from sham-operated and nonoperated nerves is as follows: RAM (4 and 14), SAM (12 and 11), D-hair (4 and 7), AM (8 and 18), and C fibres (4 and 7). Data are expressed as means ± SEM. N, number of fibres.

**Figure 4:**
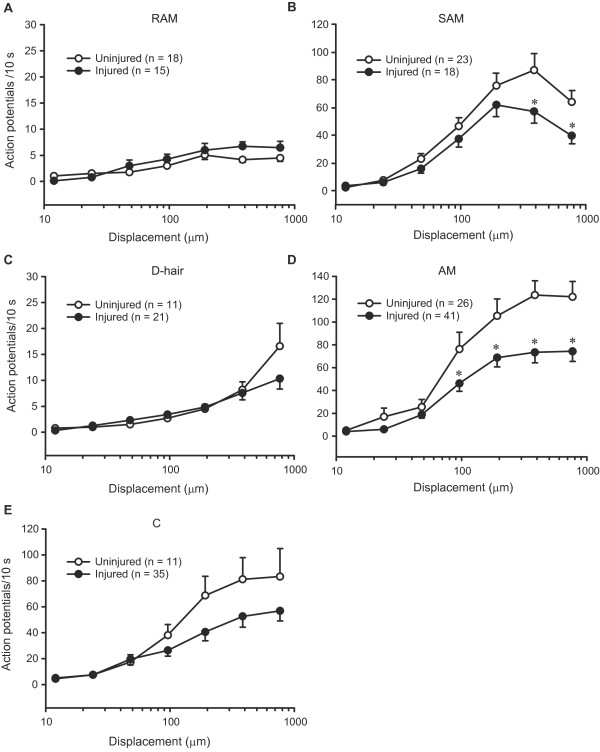
**Discharges of sensory fibres to mechanical stimulation following nerve injury.** Discharge rates to nanomotor stimulation were not significantly altered in RAM (Aβ), D-hair (Aδ) and C fibres (*P* > 0.05; 2-way RM ANOVA) (**A, C, E**), but were diminished in SAM (Aβ) and AM (Aδ) fibres in injured nerves (*P < 0.05, compared to uninjured nerves; 2-way RM ANOVA, Bonferroni *t* test) (**B, D**). In uninjured nerves, the number of fibres from sham-operated and nonoperated nerves is the same as in Figure 3. All data are expressed as means ± SEM. N, number of fibres.

**Figure 5:**
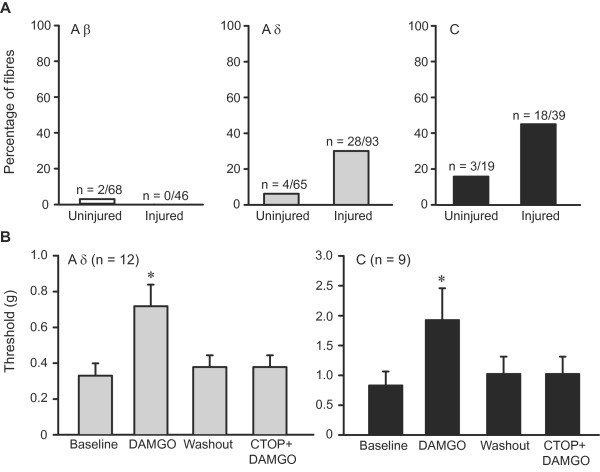
**Effects of DAMGO on mechanical thresholds of sensory fibres following nerve injury.** (**A**) Percentages of DAMGO-responding fibres in uninjured and injured nerves. The number of Aδ and C, but not of Aβ, fibres in which DAMGO (100 μM) increased von Frey thresholds was significantly higher in injured compared to uninjured nerves (*P* < 0.05; Fisher exact test for C fibres, and chi-square test for Aβ and Aδ fibres; calculated on raw data). In uninjured nerves, the number of fibres from sham-operated and nonoperated nerves is the same as in Figure 2. (**B**) Elevation of mechanical thresholds of Aδ and C fibres in injured nerves by DAMGO (100 μM), and its blockade by DAMGO washout or pre-treatment with μ-opioid receptor antagonist CTOP (100 μM). All Aδ fibres were classified as AM nociceptors. *P < 0.05, compared to all other conditions (1-way RM ANOVA, Bonferroni *t* test for Aδ fibres, and 1-way RM ANOVA on ranks, Tukey test for C fibres). Data are expressed as means ± SEM. N, number of fibres.

We investigated myelinated Aβ and Aδ fibres, and unmyelinated C fibres from injured (2 weeks after CCI) and uninjured saphenous nerves in an *in vitro* skin-nerve preparation. Uninjured nerves comprised nonoperated and sham-operated nerves, 2 weeks after operation (Figure 2, Figure 3, Figure 4, Table 1 and Figure 5A). They were pooled, as we did not observe significant differences between the two conditions in the fibres’ conduction velocities, thresholds, latencies and discharge rates to mechanical stimulation (*P* > 0.05; data not shown).

**Table 1:** Effects of DAMGO on mechanical thresholds of sensory fibres in uninjured and injured nerves

**Fibre type**	** Group**	**Uninjured nerves**			**Injured nerves**		
		**Numbers**	**Baseline (g)**	**DAMGO (g)**	**Numbers**	**Baseline (g)**	**DAMGO (g)**
**Aβ**	All fibres	n = 68	0.14 ± 0.02	0.15 ± 0.02	n = 46	0.11 ± 0.02	0.11 ± 0.02
	DAMGO-responders	n = 2	0.22 ± 0.09	0.42 ± 0.12	n = 0	-	-
	DAMGO-nonresponders	n = 66	0.14 ± 0.02	0.14 ± 0.02	n = 46	0.11 ± 0.02	0.11 ± 0.02
**Aδ**	All fibres	n = 65	0.35 ± 0.05	0.38 ± 0.05	n = 93	0.40 ± 0.04	0.65 ± 0.08 *
	DAMGO-responders	n = 4	0.25 ± 0.11	0.69 ± 0.13	n = 28	0.38 ± 0.06	1.19 ± 0.23
	DAMGO-nonresponders	n = 61	0.35 ± 0.05	0.35 ± 0.05	n = 65	0.44 ± 0.07	0.44 ± 0.07
**C**	All fibres	n = 19	0.61 ± 0.14	0.72 ± 0.17	n = 39	0.64 ± 0.09	1.03 ± 0.18 *
	DAMGO-responders	n = 3	0.98 ± 0.15	1.69 ± 0.40	n = 18	0.70 ± 0.13	1.56 ± 0.31
	DAMGO-nonresponders	n = 16	0.54 ± 0.15	0.54 ± 0.15	n = 21	0.59 ± 0.12	0.59 ± 0.12

DAMGO was applied at a concentration of 100 μM. N represents the number of fibres, and g refers to the fibre thresholds. *P < 0.05, compared to the respective baseline thresholds (Wilcoxon test). No statistical evaluation was performed for DAMGO-responders or DAMGO-nonresponders in uninjured or injured nerves. In uninjured nerves, the number of fibres from sham-operated and nonoperated nerves is the same as in Figure 2. Data are expressed as means ± SEM.

First we classified sensory fibres based on their conduction velocity. Fibres conducting > 10 m/s were assigned to Aβ, those conducting 1.2 – 10 m/s to Aδ and those with conduction velocities < 1.2 m/s were classified as C fibres, according to Koltzenburg et al. [29]. Conduction velocities of Aβ and Aδ fibres were slightly decreased in injured compared to uninjured nerves (*P* < 0.05), while those of C fibres were not significantly altered by the injury (*P* > 0.05; Figure 2A).

To examine the mechanosensitivity of sensory fibres, we assessed their thresholds with calibrated von Frey hairs applied to the fibres’ receptive fields. Additionally, we evaluated the response properties of some Aβ, Aδ and C fibres to increasing mechanical stimuli evoked by the nanomotor, a computer-controlled mechanical stimulator. Based on the discharge pattern, Aβ fibres were further divided into rapidly adapting mechanoreceptors (RAM) and slowly adapting mechanoreceptors (SAM), whereas Aδ fibres were divided into slowly adapting Aδ mechanonociceptors (AM) and rapidly adapting down-hair (D-hair) fibres [13]. D-hair fibres are also characterized by very low mechanical thresholds (the majority already responds to the lowest von Frey hair, i.e. ~ 0.007 g) and relatively large receptive fields [29]. All C fibres tested displayed a slowly adapting discharge pattern, their firing rate increased progressively to increasing mechanical stimulation (nanomotor), and they revealed mechanical thresholds in the range of 0.07 – 4.5 g, indicating they were mainly nociceptors. In contrast, low-threshold C fibres were described to respond to von Frey hairs of ≤ 0.25 mN (or ~ 0.02 g) in rats [30] or to very fine von Frey hairs in mice (0.07 mN or ~ 0.007 g) [31]. The mechanical thresholds of Aβ, Aδ and C fibres were not significantly changed by the nerve injury (*P* > 0.05; Figure 2B). Similarly, the thresholds of the Aβ and Aδ subpopulations did not significantly differ between uninjured and injured nerves: RAM (0.07 ± 0.02 g [n = 18] vs. 0.07 ± 0.02 g [n = 15]), SAM (0.18 ± 0.03 g [n = 23] vs. 0.15 ± 0.04 g [n = 18]), D-hair (0.007 ± 0 g [n = 11] vs. 0.017 ± 0.004 g [n = 21]) and AM (0.36 ± 0.05 g [n = 26] vs. 0.54 ± 0.06 g [n = 41]) (*P* > 0.05).

There is evidence that some A nociceptive afferents conduct in the Aβ fibre conduction velocity range. As the sensory properties of Aδ and Aβ nociceptive neurons seem comparable [32], we attempted to classify Aβ nociceptive fibres based on a “high mechanical threshold” that is within the range of Aδ (AM) nociceptors (i.e. a mean threshold of ≥ 0.3 g; see above). Such threshold (range 0.3 – 0.54 g) was revealed by 18 of 68 Aβ fibres (27%) in uninjured nerves, and by 10 of 61 Aβ fibres (16%) in injured nerves presented in Figure 2. Within the Aβ fibre subpopulations, similar thresholds were revealed by 10 of 23 SAM fibres (44%) in uninjured nerves, and by 4 of 18 SAM fibres (22%) in injured nerves. We did not find “high threshold” RAM fibres (except for 1 RAM fibre in uninjured nerve, which had a threshold of 0.3 g). All remaining fibres had lower mechanical thresholds (range 0.007 – 0.13 g). The proportion of “high-threshold” Aβ fibres was not significantly different between injured and uninjured nerves (*P* > 0.05). The 27% of “high-threshold” Aβ fibres we estimated in uninjured nerves is comparable to a result of a previous study reporting ~ 25% of A fibre nociceptors conducting in the Aβ fibre velocity range in the naïve mouse saphenous nerve ([29]; see also [32]).

Next, we assessed the latency and the discharge rate of each fibre type to mechanical stimulation with the nanomotor. The relationship between the latency and the mechanical stimulus strength was very characteristic for fibre types. In uninjured nerves, rapidly adapting RAM and D-hair fibres had very short latencies, and the shortest were found at the lowest mechanical stimuli. The latencies of these fibres increased with increasing mechanical stimuli (Figure 3A,C), possibly as a result of a desensitisation to repetitive stimulation, as discussed earlier [13]. By contrast, mechanical latencies of slowly adapting SAM, AM, and C fibres were initially very long, but shortened to a plateau as stimulus strength increased. They had the shortest latencies at higher mechanical stimulations (Figure 3B,D,E), in line with the study by Milenkovic et al. [13]. There were no significant differences in mechanical latencies of RAM, SAM, D-hair, AM and C fibres between uninjured and injured nerves (*P* > 0.05; Figure 3).

Rapidly and slowly adapting fibre types also displayed characteristic discharge rates. In uninjured nerves, RAM and D-hair fibres had low discharge rates, slightly increasing with higher mechanical stimuli (Figure 4A,C). In contrast, SAM, AM and C fibres displayed a clear increase in discharge rates with increasing stimulus strength (Figure 4B,D,E), although the discharge rates of SAM fibres tended to decrease at the highest mechanical intensities (Figure 4B). The overall lower discharge rates of rapidly adapting compared to slowly adapting fibres results from measuring the total number of action potentials per a 10 s ramp-and-hold stimulus (see methods). Rapidly adapting fibres respond solely to the (shorter-lasting) ramp phase of the stimulus, whereas slowly adapting fibres respond to the (longer-lasting) hold phase [13].

Interestingly, although all fibre types in injured nerves displayed a similar discharge characteristic, the injury differentially affected the frequency of their discharges. Thus, the discharge rates significantly dropped following injury at higher mechanical displacements (384 μm and 768 μm) in SAM (*P* < 0.05; Figure 4B), and even more evidently in AM fibres (at 96 μm, 192 μm, 384 μm and 768 μm; *P* < 0.05; Figure 4D). The discharge rates tended to decrease also in C fibres albeit not significantly (*P* > 0.05; Figure 4E). RAM and D-hair fibres revealed no significant differences in their discharge rates following injury (*P* > 0.05; Figure 4A,C).

Collectively, nerve injury slightly lowered the conduction velocities of myelinated Aβ and Aδ fibres. However, it did not affect the mechanical thresholds and latencies of any fibres, while the firing rates of SAM and AM fibres were reduced. This indicates that sensory fibres in injured nerves were not sensitised to mechanical stimuli with respect to their thresholds, latencies and discharge rates.

### μ-Opioid elevates mechanical thresholds of cutaneous Aδ and C nociceptors in injured nerves

We then investigated the effect of μ-opioid receptor agonist [D-Ala^2^,*N*-Me-Phe^4^,Gly^5^]-ol-enkephalin (DAMGO; 100 μM) on mechanical von Frey thresholds of the fibres. This dose was the most effective (of 1 – 500 μM) in our pilot experiments. Analysis of all fibres tested with DAMGO in uninjured nerves revealed no overall statistically significant differences in the mechanical thresholds of Aβ, Aδ and C fibres after DAMGO application compared to the baseline thresholds (*P* > 0.05; see “All fibres” in Table 1). In contrast, analysis of all fibres tested with DAMGO in injured nerves revealed a statistically significant elevation in mechanical thresholds of Aδ and C fibres (*P* < 0.05), but not of Aβ fibres (*P* > 0.05), following DAMGO application (see “All fibres” in Table 1). Among all fibres tested we identified fibres that responded with increased thresholds by at least one von Frey hair force following DAMGO application. This was the lowest change we could measure. Due to the logarithmic scale of the von Frey hairs’ force, such change represents a nearly 2-fold or higher increase in the mechanical thresholds. Possible subtle changes were therefore not detected. Fibres which did not respond were considered DAMGO-nonresponders (Table 1). DAMGO did not decrease the fibres’ thresholds. In uninjured nerves, very few Aβ (~ 3%), Aδ (~ 6%) and C fibres (~ 15%) revealed elevated thresholds by DAMGO. In sharp contrast, ~ 30% of Aδ and ~ 46% of C fibres, but none of Aβ fibres, responded to DAMGO with elevated thresholds in injured nerves. The 28 DAMGO-responding Aδ fibres in injured nerves had a mean mechanical threshold of 0.38 ± 0.06 g (range 0.04 – 1.2 g), indicating they were AM nociceptors (Figure 5A). The number of DAMGO-responding Aδ and C fibres was significantly higher following nerve injury (*P* < 0.05; Figure 5A).

Therefore, the receptor specificity of the effect was investigated in DAMGO-responding Aδ and C fibres in injured nerves (Figure 5B). We found that the elevated von Frey thresholds of Aδ and C fibres following DAMGO application (*P* < 0.05) were reversed by DAMGO washout (*P* < 0.05), and prevented by pre-treatment of the fibres’ receptive fields with the selective μ-opioid receptor antagonist Cys^2^-Tyr^3^-Orn^5^-Pen^7^-amide (CTOP; 100 μM) (*P* < 0.05). The thresholds after washout or pre-treatment with CTOP were not significantly different from baseline thresholds (*P* > 0.05; Figure 5B). All Aδ fibres in Figure 5B responded throughout the static phase of the nanomotor stimulation (i.e. were slowly adapting) and had localized receptive fields, classifying them as AM nociceptors. D-hair fibres did not respond to DAMGO. CTOP (100 μM) alone did not significantly change the thresholds of Aδ (0.52 ± 0.1 g before and after CTOP; n = 11) and C fibres (0.64 ± 0.1 g before vs. 0.61 ± 0.1 g after CTOP; n = 10) (*P* > 0.05), tested in a separate group of fibres in injured nerves. Likewise, control buffer (100 μl) did not significantly alter the thresholds of Aδ (0.45 ± 0.09 g before vs. 0.46 ± 0.09 g after buffer; n = 32) and C fibres (0.79 ± 0.2 g before vs. 0.8 ± 0.19 g after buffer; n = 11) (*P* > 0.05). Together, these results clearly show that DAMGO activates μ-opioid receptors in cutaneous C and Aδ (AM) nociceptors, and elevates their mechanical thresholds following nerve injury.

### μ-Opioid diminishes discharges to mechanical stimulation in C nociceptors in injured nerves

**Figure 6:**
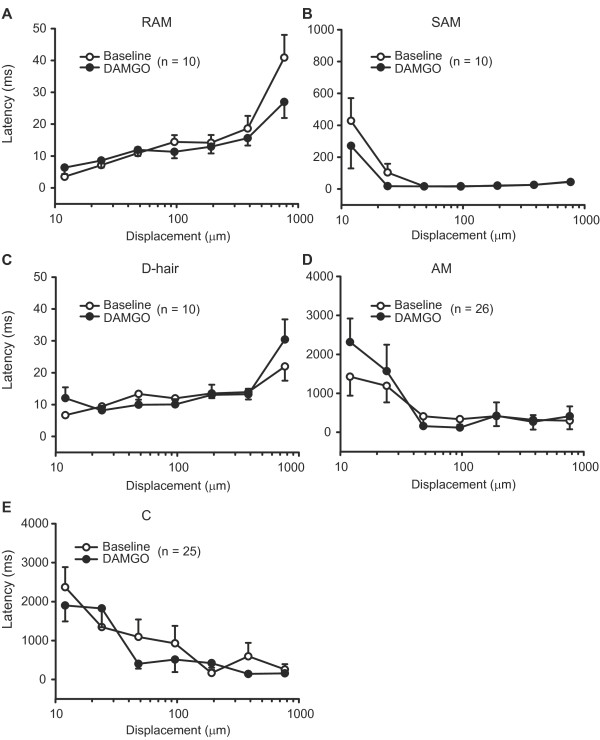
**Effects of DAMGO on mechanical latencies of sensory fibres in injured nerves.** Baseline mechanical latencies of Aβ (RAM, SAM), Aδ (D-hair, AM) and C fibres to nanomotor stimulation were not significantly changed by DAMGO (100 μM) (*P* > 0.05; 2-way RM ANOVA) (**A-E**). Data are expressed as means ± SEM. N, number of fibres.

DAMGO (100 μM) did not significantly change the mechanical latencies of RAM, SAM, D-hair, AM and C fibres in uninjured nerves (*P* > 0.05; data not shown), nor did it affect the latencies of RAM, SAM and D-hair fibres in injured nerves (*P* > 0.05; Figure 6A-C). To find out whether the effects of DAMGO on von Frey thresholds of AM and C fibres in injured nerves correlate with possible effects on latencies and discharges, we analysed the data from all AM and C fibres tested, and separately only from AM and C fibres in which DAMGO elevated the thresholds. This was not necessary for RAM and SAM fibres, because DAMGO did not alter their thresholds (see Aβ fibres in Table 1 and Figure 5A), similar to D-hair fibres (see above). Of 26 AM fibres tested with the nanomotor (Figure 6D), 7 AM fibres had elevated von Frey thresholds after DAMGO (0.47 ± 0.1 g before vs. 1.05 ± 0.3 g after DAMGO). Of 25 C fibres tested (Figure 6E), 10 C fibres had increased mechanical thresholds after DAMGO (0.66 ± 0.1 g before vs. 1.58 ± 0.4 g after DAMGO). Nevertheless, the latencies of AM and C fibres were unaltered by DAMGO (*P* > 0.05), regardless whether the data were analysed from all fibres tested (Figure 6D,E) or only from fibres in which DAMGO elevated the thresholds (data not shown). There were also no significant effects of control buffer in injured nerves in all fibre types (*P* > 0.05; data not shown).

**Figure 7:**
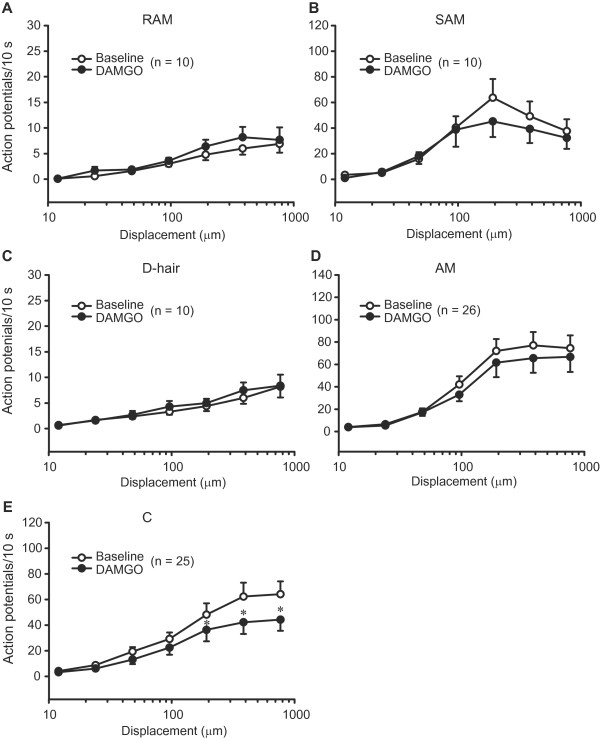
**Effects of DAMGO on discharges of sensory fibres in injured nerves.** Baseline discharge rates of Aβ (RAM, SAM) and Aδ (D-hair, AM) fibres to nanomotor stimulation were not significantly altered by DAMGO (100 μM) (*P* > 0.05; 2-way RM ANOVA) (**A-D**). DAMGO significantly diminished the discharges of C fibres (*P < 0.05, 2-way RM ANOVA, Bonferroni *t* test) (**E**). All data are expressed as means ± SEM. N, number of fibres.

**Figure 8:**
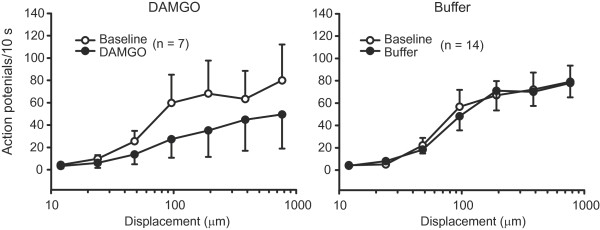
**Effects of DAMGO on discharges of AM (Aδ) fibres with elevated mechanical thresholds in injured nerves.** Baseline discharge rates of AM nociceptors to the nanomotor stimulation were not significantly changed by DAMGO (100 μM) or control buffer (*P* > 0.05; 2-way RM ANOVA). Data are expressed as means ± SEM. N, number of fibres.

The discharge rates were evaluated analogously in the same fibres. DAMGO did not affect the discharge rates of any fibres in uninjured nerves (*P* > 0.05; data not shown). It did not significantly modify the discharge rates of RAM, SAM, D-hair and AM fibres in injured nerves either (*P* > 0.05; Figure 7A-D). There was a tendency to lower firing rates of AM fibres with elevated thresholds (n = 7; see above), although the effect was not significant (*P* > 0.05; Figure 8). Control buffer had no effect on the discharge rates of AM fibres (*P* > 0.05; Figure 8).

**Figure 9:**
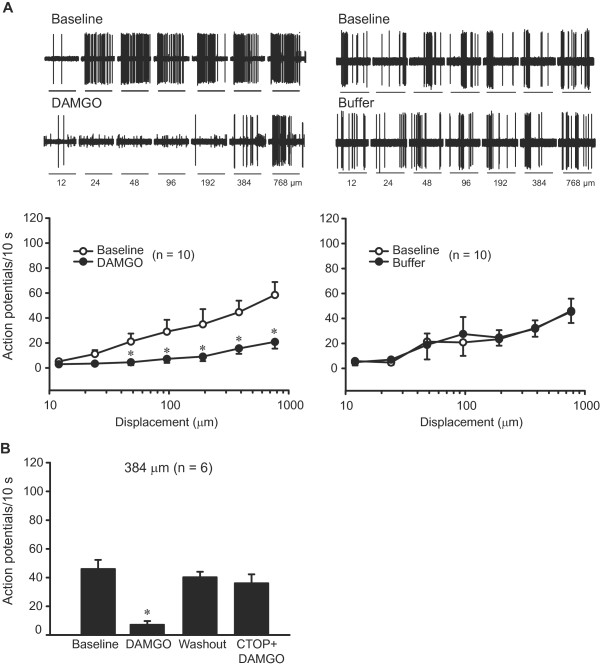
**Effects of DAMGO on discharges of C fibres with elevated mechanical thresholds in injured nerves.** (**A**) Representative examples of C fibre firing (upper panels) and quantitative analysis (lower panels) showing that baseline discharge rates of C nociceptors to nanomotor stimulation were diminished by DAMGO (100 μM) (*P < 0.05; 2-way RM ANOVA, Bonferroni *t* test), but not by control buffer (*P* > 0.05; 2-way RM ANOVA). DAMGO and buffer were tested on separate sets of fibres. In upper panels the displacements are marked with dark lines, and the interstimulus sequences are removed. (**B**) DAMGO (100 μM)-induced reduction in the discharge rate was reversed by DAMGO washout and prevented by pre-application of CTOP (100 μM). This was tested in additional C fibres at a single nanomotor stimulation (384 μm). ^∗^*P* < 0.05, compared to all other conditions (1-way RM ANOVA; Bonferroni *t* test). All data are expressed as means ± SEM. N, number of fibres.

In contrast, C fibre discharge rates were significantly reduced by DAMGO (at 192 μm, 384 μm and 768 μm; *P* < 0.05; Figure 7E). Apparently, this effect was attributed to C fibres in which DAMGO elevated von Frey thresholds (n =10; see above) because a separate analysis of only these fibres showed robustly diminished discharge rates following DAMGO at the majority of displacements (48 μm, 96 μm, 192 μm, 384 μm and 768 μm; *P* < 0.05; Figure 9A). The discharge rates of C fibres returned to baseline following DAMGO washout (*P* > 0.05; data not shown). The data after washout were evaluated in 7 of 10 C fibres depicted in Figure 9A (lower panel), and it was not possible to run the nanomotor a fourth time to assess the effect of pre-treatment with CTOP, likely because of multiple repetitive stimulations (see methods).

Therefore, the effect of CTOP was tested in 6 (of total 11) additional C fibres at only one displacement (384 μm; Figure 9B). The DAMGO-induced decrease in the discharge rates of these C fibres (*P* < 0.05) was reversed by DAMGO washout (*P* < 0.05) and prevented by CTOP (*P* < 0.05). The effects of washout and CTOP were not significantly different from the baseline (*P* > 0.05; Figure 9B). CTOP (100 μM) alone did not significantly change the discharges of C fibres (38.7 ± 6.44 before vs. 33.8 ± 6.38 after CTOP; n = 10) (*P* > 0.05), tested in a separate group of fibres in injured nerves, at the 384 μm displacement. Control buffer had no effect on the discharge rates of C fibres (Figure 9A) and of RAM, SAM and D-hair fibres (data not shown) in injured nerves (*P* > 0.05). Together, DAMGO did not affect the mechanical latencies of sensory fibres, but it diminished the firing of C fibres in damaged nerves.

## Discussion

Our findings suggest that primary sensory fibres are not overtly sensitised to mechanical stimuli following neuropathy. However, nerve injury renders nociceptors responsive to μ-opioid receptor agonist. Inhibition of action potential generation or propagation in nociceptors might represent a possible mechanism that underlies peripheral opioid-mediated alleviation of mechanical hypersensitivity in neuropathic conditions.

### Nerve injury and mechanical sensitivity of peripheral sensory fibres

The primary afferents we tested were most likely spared because the fibres do not fully regenerate 2 weeks after nerve damage [33]. Reduction in the conduction velocities of Aβ and Aδ, but not C fibres, in injured nerves suggests that we were recording from A fibres with myelin damage, observed in neuropathy [34]. Earlier studies found a decreased conduction velocity of C or Aδ fibres following CCI of the sciatic nerve [15,35], or no changes after spinal nerve ligation [7]. A variable degree of the injury defined, for example, by the ligature tightness around the nerves might account for these differences.

We did not detect changes in the mechanical thresholds and latencies of any fibre type following CCI. Interestingly, the discharges of SAM and AM fibres were substantially decreased, and there was a tendency to such effect in C nociceptors from injured nerves. This was probably not related to the repetitive stimulation per se because we did not find such changes in RAM and D-hair fibres. In contrast to our findings, microneurographic studies in patients with neuropathic pain of diverse aetiology reported reduced mechanical thresholds of C fibres, although there was no direct comparison to control patients, and data are based on recordings from a small number of fibres in few patients [36-38]. In animal models of diabetic neuropathy, Aδ fibres displayed decreased thresholds and increased discharges to mechanical stimulation, whereas C fibres responded with enhanced firing but unaltered thresholds [39-42]. In models of traumatic neuropathy, a spinal nerve ligation and saphenous nerve transection reduced the thresholds or increased the firing of C or Aδ fibres to mechanical stimuli [6,7]. Conversely, C fibres revealed elevated mechanical thresholds after CCI of the saphenous nerve [15]. Similar to our results, unchanged C and A nociceptor thresholds or lower SAM fibre discharges were reported following nerve transection or partial ligation [6,14]. Collectively, the diversity of neuropathic conditions makes it difficult to draw a clear conclusion on nociceptor mechanosensitivity. It appears, however, that traumatic neuropathy does not overtly sensitise skin-innervating nociceptors to mechanical stimulation, in line with our findings.

Nevertheless, it is possible that mechanical sensitisation occurred in a different form (e.g. enhanced afterdischarges) [39] and/or in a subset of sensory afferents that we did not identify (see for example [43]). It also could arise in nociceptors from neighbouring uninjured nerves [44] or could be acquired by previously mechanoinsensitive afferents [45]. The latter fibres could not be tested in our experiments because mechanical rod probing as a search stimulus prevented their inclusion.

Conversely, it is likely that central sensitisation is more relevant to neuropathy-induced mechanical hypersensitivity *in vivo*[46]. It can be triggered by spontaneous ectopic activity in nociceptive afferents [7,44,47]. We observed spontaneous activity in primary afferent nociceptors, however, we hardly detected cutaneous receptive fields of these fibres, suggesting that it originated at the nerve injury site, as reported previously [47,48]. Moreover, nerve damage reduces primary afferent-evoked inhibitory (GABA) postsynaptic currents [49]. Together, even if primary nociceptive fibres in injured nerves are not sensitised to mechanical stimuli, the enhanced central responses to peripheral nociceptive input could result in an augmented perception of mechanical stimulation *in vivo*.

### Peripheral μ-opioid receptors and mechanical sensitivity of cutaneous nociceptors following nerve injury

We demonstrate that DAMGO applied on cutaneous receptive fields substantially elevated the mechanical thresholds of Aδ (AM) and C nociceptors and diminished the firing rate of C fibres in injured nerves. The effects of DAMGO were reversed by its washout and were prevented by pre-application of CTOP, confirming specific actions through μ-opioid receptors. Whereas hypersensitivity to innocuous dynamic mechanical stimuli (“allodynia”) is proposed to be mediated by large myelinated afferents [50] and/or low-threshold C mechanoreceptors [31], responses to static pressure stimuli (static mechanical hyperalgesia) seem to be mediated by primary Aδ and/or C afferents in animals [51,52] and humans [1] under neuropathic pain conditions, suggesting possible clinical implication of our findings.

DAMGO elevated the mechanical thresholds in 30 – 46% of Aδ and C fibres in injured nerves. This corresponds with the percentage of DRG neurons expressing μ-opioid receptor protein [19]. In line with this, DRG neurons that did not respond to DAMGO in patch clamp recordings exhibited little or no μ-opioid receptor mRNA [53]. Thus, fibres in which DAMGO did not increase thresholds in our study likely represent neurons that do not express μ-receptors. Furthermore, our results revealed a correlation between DAMGO-induced elevated mechanical thresholds and decreased discharge rates in case of C fibres, but not AM fibres. The lack of a significant change in the discharge rate of AM fibres might be related to the relatively high response variability (Figure 8). Alternatively, the effects of DAMGO on the thresholds might be not predictive for its actions on the firing of AM nociceptors. Following inflammation or irradiation of the skin, opioids also more efficiently suppressed the firing of C than Aδ fibres [10,54]. Furthermore, we did not observe significant alterations in the responses of low threshold D-hair and Aβ (RAM and SAM) fibres after DAMGO application. Similarly, morphine applied on the spinal cord attenuated C fibre-, but not Aβ fibre-evoked spinal neuron responses after spinal nerve ligation [55].

In uninjured nerves, very few Aβ, Aδ and C fibres had increased mechanical thresholds and none had altered latencies and discharges following DAMGO application. This is in agreement with previous recordings in naïve nerves [10,54]. Also, *in vivo* behavioural studies reported absence [18,20] or very week [25] antinociceptive effects of opioids injected into contralateral, uninjured paws of animals with neuropathy. In fact, there is substantial evidence that peripheral analgesic effects of opioids are significantly more pronounced in injured than in uninjured tissue, which was most extensively studied in inflammation [17,56]. Some authors proposed that processes characteristic for inflammatory pain (e.g. immune cells and their mediators, perineurial barrier disruption) are relevant to neuropathic pain as well [26]. However, immune cells accumulate at the trunk of injured nerves but usually not in paws innervated by these nerves [24,56]. Also, although μ-opioid receptors were up-regulated in the paw skin following CCI, they were not identified on nerve terminals [57], and their coupling and signalling has not been investigated so far. Additionally, while the blood-nerve barrier was disrupted at the nerve injury site, this was not examined in paws [58]. Thus, although there is no doubt that opioid receptors at the peripheral terminals of proximally injured nerves are functional (our current findings; see also below), the underlying mechanisms await exploration.

Opioid treatment of neuropathic pain is limited by serious side effects mediated by opioid receptors in the CNS [16]. In inflammatory conditions this was overcome by selective activation of peripheral opioid receptors, in animals and humans [17,56]. Exploring a similar approach in neuropathic pain patients is supported by a promising clinical pilot trial [28] and numerous behavioural studies reporting attenuation of mechanical hypersensitivity in animal models of neuropathy. Antinociceptive effects have been found following systemic injection of classical and peripherally-restricted opioids [18,22,23] as well as after application of opioids, including DAMGO, in systemically inactive doses into hind paws innervated by the damaged nerves [20-22,25-27]. Clearly, despite substantial behavioural evidence, there is a need for mechanistic approaches addressing such peripheral actions of opioids in neuropathy.

## Conclusions

We have demonstrated that primary nociceptive fibres in injured nerves are not sensitised to mechanical stimuli with respect to the thresholds, latencies and discharge rates. However, enhanced central responses to a peripheral nociceptive input possibly result in an augmented mechanosensation *in vivo*. Activation of μ-opioid receptors in cutaneous nociceptors in injured nerves elevates the thresholds and diminishes the firing as a consequence of inhibition of nociceptor action potential generation or propagation. This likely prevents an increased central transmission and might represent a possible mechanism underlying the opioid-mediated improvement of mechanohypersensitivity in painful neuropathies.

## Methods

### Ethical approval

Experiments were performed according to the guidelines of the International Association for the Study of Pain [59], and were approved and governed by the state animal care committee (Landesamt für Gesundheit und Soziales, Berlin).

### Animals and surgeries

Experiments were performed in male C57BL/6J mice (6 – 8 weeks old) bred at the Charité, Campus Benjamin Franklin, Berlin. Animals were housed in groups of 6 per cage lined with ground corncob bedding. They were kept on a 12 h light/dark schedule with food pellets and water *ad libitum*. Room temperature was 22 ± 0.5°C and the relative humidity was 60 – 65%.

Chronic constriction injury of the saphenous nerve was performed in deeply anesthetised mice. Animals were placed in a glass chamber on a perforated ceramic plate, which was located above tissues soaked with approximately 15 ml of isoflurane (Abbott, Wiesbaden, Germany), until anaesthesia was initiated. Subsequently, the animal’s nose was covered by a tube attached to an anaesthesia machine (Aestiva 3000, Datex-Ohmeda, GE Healthcare) delivering a gaseous mixture of isoflurane (3 – 4%) and oxygen throughout the procedure. The saphenous nerve was exposed at the level of the right thigh, and three nylon sutures (8–0) were loosely tightened with about 1 mm spacing around the nerve [57]. The wound was closed using nylon sutures. Sham operation was performed by exposing but not ligating the nerve.

### Nociceptive testing

Animals (n = 6 per surgery type) were habituated to the test cages with wire-mesh floor daily, starting 6 days prior to the testing. Few hours before and 2 weeks after CCI or sham operation the withdrawal thresholds of the hind paws were determined with calibrated von Frey hairs (Stoelting, Wood Dale, IL). In the same groups of mice, von Frey hairs were applied to the plantar glabrous skin and 1.5 – 2 h later to the dorsal hairy skin of the hind paw, according to an up-down method [60]. Testing began with a 0.4 g hair. If the mouse withdrew the paw the next weaker hair was applied. In case of no withdrawal the next stronger hair was applied. The maximal number of applications was 6 – 9 and the cut-off was 4 g, as previously [24]. The examiner was unaware of the surgery type. After completion of experiments mice were killed with an overdose of isoflurane.

### Skin-nerve preparation and electrophysiology

The skin-nerve preparation was used as previously described [29]. Naïve mice and those at 2 weeks after CCI or sham surgery were killed with an overdose of isoflurane. The examiner could not be blinded to the surgery type due to the visible ligatures following CCI in the dissected injured nerves. The shaved skin of the lower hind limb, predominantly including the hairy skin of the paw, and the saphenous nerve in continuity were dissected free and placed with the corium side up in an organ bath. The skin with a part of the nerve including the CCI site was placed in one chamber which was perfused with a synthetic interstitial fluid (SIF; ~ 30°C). Its composition was (in mM): NaCl 123, KCL 3.5, MgSO_4_ 0.7, NaH_2_PO_4_ 1.5_,_ CaCl_2_ 2, sodium gluconate 9.5, glucose 5.5, sucrose 7.5, HEPES 10, at a pH of 7.4. The remaining part of the nerve was placed in a second, adjacent chamber filled with mineral oil for fibre teasing and single-unit recordings. The perineurium was removed and small bundles of fibres were teased and attached to the recording electrode. The receptive field of each fibre was identified by a mechanical search stimulus (glass rod) applied to the paw skin. Its location in relation to the nerve and blood vessels helped to apply mechanical stimuli to the same site during subsequent stimulations. Action potentials from each fibre were recorded extracellularly with a low-noise amplifier (Digitimer, Hertfordshire, UK), visualized on an oscilloscope and on a computer with the help of a digital converter (PowerLab 4/26, ADInstruments, Oxfordshire,UK), and analyzed off-line using the LabChart (version 6, ADInstruments) spike histogram extension software. Single fibres were classified by conduction velocity and shape and width of the action potential, and by adaptation properties to constant mechanical stimulation. The conduction velocity was determined by electrically stimulating the fibre’s receptive field with suprathreshold current pulses (in the range of 1 – 10 mA) with durations of 50, 150 or 500 μs using a sharp tungsten metal electrode. The conduction velocity was calculated as the distance (in mm) from the receptive field to the recording electrode, divided by the electrical latency (in ms) of the action potential [29]. Next, the mechanical threshold of each fibre was estimated by evoking action potentials to calibrated von Frey hairs (Stoelting). The hairs of the following forces were used: 0.007, 0.008, 0.042, 0.072, 0.13, 0.3, 0.54, 0.82, 1.27, 2.5 and 4.5 g. Testing began using a 0.13 g hair. If the fibre responded the just preceding weaker hair was applied. In case of no response the next stronger hair was applied. The number of applications was 3 – 7, with an interval of 10 – 20 s between individual von Frey hairs [40]. The threshold was defined as the force (in grams) of the smallest von Frey hair necessary to evoke at least one action potential. Most fibres were subsequently tested with a computer-controlled mechanical stimulator (nanomotor; Kleindiek, Reutlingen, Germany) equipped with a stainless metal probe. The nanomotor was used to apply ramp-and-hold displacement stimuli to the skin. The ramp phase of the stimulus had a constant velocity (2.9 mm/s) throughout the stimulation protocol. Standardised increasing displacement stimuli (12, 24, 48, 96, 192, 384 and 768 μm) of 10 s duration were applied to the receptive field at regular intervals (30 s). Before each stimulation protocol, the nanomotor probe was positioned above the receptive field. Using small movements (96 μm) it was advanced onto the receptive field until one action potential was evoked. Then the probe was moved 96 μm upwards again. The same approach was applied while stepwise reducing the movement to the smallest stimulus (12 μm) used in the nanomotor stimulation protocol. The starting position of the probe was therefore just above the threshold for each recorded unit. The nanomotor was moved only vertically allowing its positioning at the same site each time. The discharge rate was defined as the number of action potentials per 10 s. The mechanical latency (in ms) was defined as the latency between the onset of the mechanical stimulus and the first action potential corrected for conduction delay, and was measured for each mechanical displacement. Both parameters were calculated off-line with the spike histogram extension software.

### Drug treatment in the skin-nerve preparation

Drugs were applied in SIF buffer (100 μl; ~ 30°C). The effects of DAMGO (100 μM) on von Frey thresholds of sensory fibres were assessed as follows: After determination of the fibre’s baseline threshold, a metal ring (6 mm in diameter) was sealed over the receptive field and the SIF buffer inside the ring was replaced with DAMGO for 2 min. Afterwards, DAMGO was taken out, the ring was removed and the fibre’s von Frey threshold was re-evaluated. Next, the fibre’s receptive field was washed for 10 – 15 min [10], and the threshold was assessed a third time. If the threshold was elevated by DAMGO, its μ-opioid receptor selectivity was tested with the antagonist CTOP (100 μM). In this case, the ring was again sealed (after DAMGO washout), filled for 3 min with CTOP which was then replaced with DAMGO for 2 min. Subsequently, DAMGO was taken out, the ring was removed and the von Frey threshold was tested a fourth time. As a control treatment, SIF buffer (100 μl) was tested accordingly on a separate group of fibres.

We also investigated the effects of DAMGO on the discharge rates and the mechanical latencies of sensory fibres using the nanomotor. After determining the fibre’s mechanical threshold with von Frey hairs, the computer-controlled nanomotor protocol was run, as described above. DAMGO or SIF buffer were applied to the receptive field for 2 min, as described above. After removal of DAMGO or buffer, the von Frey threshold was re-evaluated and the nanomotor protocol was run a second time. We also attempted to evaluate the discharge rate and mechanical latency following DAMGO washout and pre-application of CTOP, however, only some fibres were successfully stimulated a third time with the nanomotor protocol (i.e. after DAMGO washout; 21 stimulations), as observed previously [13]. This was likely related to multiple repetitive stimulations. Therefore, the effects of DAMGO washout and CTOP pre-treatment on the discharge rate were accordingly tested in additional C fibres at only one nanomotor displacement (384 μm). Additionally, the effect of CTOP alone was examined in a separate group of C fibres.

### Statistical analysis

Data are expressed as means ± SEM. Two-sample comparisons were made using a *t* test for independent normally distributed data, a Mann–Whitney test for independent not normally distributed data, a paired *t* test for dependent normally distributed data and a Wilcoxon test for dependent not normally distributed data. A chi-square test and Fisher exact test were used for comparison of proportions. Multiple comparisons were evaluated with 1-way or 2-way repeated measures (RM) analysis of variance (ANOVA) followed by a Bonferroni *t* test for normally distributed data, or with 1-way RM ANOVA on ranks followed by a Tukey test for not normally distributed data. The data were defined statistically significant if *P* < 0.05. All tests were performed with SigmaPlot 11 software.

## Abbreviations

AM: Aδ mechanonociceptors; ANOVA: Analysis of variance; CCI: Chronic constriction injury; CNS: Central nervous system; CTOP: Cys^2^-Tyr^3^-Orn^5^-Pen^7^-amide; DAMGO: [D-Ala^2^,*N*-Me-Phe^4^,Gly^5^]-ol-enkephalin; D-hair: Down-hair fibre; DRG: Dorsal root ganglion; RAM: Rapidly adapting mechanoreceptor; RM: Repeated measures; SAM: Slowly adapting mechanoreceptor; SIF: Synthetic interstitial fluid.

## Competing interests

The authors declare that they have no competing interests.

## Authors' contributions

YS designed electrophysiological experiments, collected and analysed electrophysiological data, and wrote the manuscript. DL collected and analysed behavioural data, and revised the manuscript. PAH helped to design electrophysiological experiments and revised the manuscript. HM conceived the study, designed experiments and wrote the manuscript. All authors approved the final version of the manuscript.
